# A 14-year time series of marine megafauna bycatch in the Italian midwater pair trawl fishery

**DOI:** 10.1038/s41597-022-01155-2

**Published:** 2022-02-14

**Authors:** Sara Bonanomi, Fabrizio Moro, Alessandro Colombelli, Jacopo Pulcinella, Caterina Maria Fortuna

**Affiliations:** 1 Italian National Research Council (CNR), Institute of Marine Biological Resources and Biotechnologies (IRBIM), Largo Fiera della Pesca 1, 60125 Ancona, Italy; 2 Italian National Institute for Environmental Protection and Research (ISPRA), via Vitaliano Brancati 60, 00144 Rome, Italy

**Keywords:** Ecology, Marine biology

## Abstract

Fisheries bycatch is recognised as a global threat to vulnerable marine megafauna and historical data can contribute to quantify the magnitude of the impact. Here, we present a collection of three datasets generated between 2006 and 2019 by a monitoring programme on marine megafauna bycatch in one of the main Italian fisheries, the northern central Adriatic midwater pair trawl fishery. The three datasets consist of: (i) monitored fishing effort; (ii) bycatch and biological data of dolphins, sea turtles and elasmobranchs; (iii) and dolphin sightings. Some information included in these datasets has already proved to provide a unique opportunity to estimate total incidental capture of species of conservation concern and trends of their relative abundance over time in the northern - central Adriatic Sea. These datasets are expected to be considered by different end users to improve the conservation of species and fishery management approaches to assess the impact of a fishery on species of conservation concern.

## Abstract (summary)

**Table:** 

Measurement(s)	marine megafauna bycatch
Technology Type(s)	monitoring • digital curation
Factor Type(s)	temporal interval
Sample Characteristic - Organism	Tursiops truncatus • Caretta caretta • Elasmobranchs
Sample Characteristic - Environment	sea
Sample Characteristic - Location	Adriatic Sea

Machine-accessible metadata file describing the reported data: 10.6084/m9.figshare.17152577

## Background & Summary

The incidental catch of non-target species (bycatch) during fishing operations is one of the major global threats for marine megafauna, including cetaceans, sea turtles and elasmobranchs^1–5^. Since these species have different life-history traits and distribution, their populations are considered to be particularly vulnerable to direct mortality caused by fishing operations, and fisheries bycatch can contribute to their decline^6–8^. However, the ability of scientists to provide advice on potential options for management measures on vulnerable species is still limited due to data availability^9^.

Within the EU Data Collection Framework, Member States have an obligation to collect and deliver a wide range of fisheries data needed for scientific advices^10^. Data are usually recorded and stored at national or at regional level by different bodies (e.g., public institutions, Non-Governmental Organizations NGOs) in different databases. Hence, datasets are fragmented, not readily available, nor easily accessible for scientific, management and conservation purposes.

Appropriate management strategies that can mitigate the impact of fisheries on vulnerable species are urgently needed, but they require easily accessible data from systematic and independent monitoring programmes^11^. In the case of the Mediterranean Sea, many authors have documented that dolphins^12^, sea turtles^13^, sharks and rays^14,15^ interact with and are incidentally taken by different types of fisheries, including trawlers, longlines, and gillnets^16^. Nevertheless, there is little quantitative data available on historical bycatch data of this marine megafauna and, only recently, few authors have started to share time series of Mediterranean fishery data in public repositories^17–20^.

To our knowledge, this is one of the first initiatives to make public historical bycatch data of marine megafauna recorded in the most heavily impacted basin of the Mediterranean Sea^21^, the northern central Adriatic Sea. This area supports a rich and valuable marine biodiversity including marine megafauna and is subjected to a variety of sources of anthropogenic pressures, mainly intense fishing activities, eutrophication, large urban development along coastal areas, and environmental pollution^22–25^. Since the early eighties, the northern central Adriatic Sea has been intensively exploited by many fisheries, including the Italian midwater pair trawl fishery, which is one of the largest in the Mediterranean^26^. Between 2006 and 2019, an extensive monitoring programme of accidental catches of marine megafauna has been conducted on this fishery under permit issued by the Italian Ministry of Agriculture, Food and Forestry (Fishery and Aquaculture directorate), in compliance with the Italian obligations to the Council Regulation (EC) 812/2004 and the EU Data Collection Framework. The primary goal of the programme was to identify and assess the impact of fisheries bycatch on cetaceans in the Italian midwater pair trawl fishery. Then, species of conservation concern like sea turtles and elasmobranchs were also included in the monitoring activity. In this framework, long-term fishery dependent data collected by trained observers provided the most reliable information on the interaction of different vulnerable species with a specific fishing gear in the northern central Adriatic Sea.

From the data collection, a database was built, and three datasets were extracted and described in the present work. Some information included in these datasets have already proved to be useful evaluating the impact^27^ and predicting the incidental catches of elasmobranchs^28,29^ and sea turtles^30^ in the Italian midwater pair trawl fishery. The information gathered in the datasets can help to assess the extent of fisheries bycatch on different vulnerable species in the Mediterranean Sea. Hence, these datasets can be particularly helpful for understanding the ecology of these species and identifying appropriate fishery management measures.

## Methods

### Data collection

Between 2006 and 2019, an average of 13 trained observers per year monitored all fishing operations of 4392 fishing trips and collect bycatch data of cetaceans, sea turtles and elasmobranchs on board 68 pelagic trawlers >15 m of overall length (LOA) in the northern-central Adriatic Sea. For each haul, they recorded operational parameters (e.g., haul duration, time of net setting and hauling, trawling speed) and environmental variables (e.g., geographical coordinates, water depth). Bycatch specimens were measured to the nearest cm using a measuring board and weighed to the nearest gram using an electronic scale or a dynamometer for the largest specimens. For each individual, physical status was assessed by examining body condition including the presence of any injuries, bleeding, the response to external stimuli, the general activity and locomotion (Table 1).

**Table 1:** Details of variables included in the shapefiles available in the Marine Data Archive.

Variable name	Domain	Variable type	U.o.M
*ID_CELL*	All	Cell unique identifier	—
*LON*	All	Longitude	Decimal degrees
*LAT*	All	Latitude	Decimal degrees
*n_Hauls*	Fishing effort	Number of hauls	—
*fsh_hrs*	Fishing effort	Total Fishing hours/cell	Hours
*avH_Len*	Fishing effort	Average haul duration/cell	minutes
*avHspeed*	Fishing effort	Average haul speed	kn
*avDepth*	Fishing effort	Average sea depth	m
*2006:2019*	Bycatch/Dolphin sightings	Abundance/Year/cell	Number of specimens
*Lngth_c*	Bycatch	Average length/cell	cm
*Wdth_cm*	Bycatch	Average disc width/cell (batoids)	cm
*Wgth_kg*	Bycatch	Average weight/cell	kg
*F*	Bycatch	Number of females	Number of specimens
*M*	Bycatch	Number of males	Number of specimens
*Unsexed*	Bycatch	Number of unsexed specimens	Number of specimens
*Alive (Good)*	Bycatch	Specimens release conditions: Alive	Number of specimens
*Alive (Bad)*	Bycatch	Specimens release conditions: Bad	Number of specimens
*Alive (Inactive)*	Bycatch	Specimens release conditions: Inactive	Number of specimens
*Dead*	Bycatch	Specimens release conditions: Dead	Number of specimens
*Nt_rlsd*	Bycatch	Specimens release conditions: Not released	Number of specimens
*ND*	Bycatch	Specimens release conditions: Unknown	Number of specimens
*CCLM_Ln*	Bycatch/Marine turtles	Minimum Curved Carapace Length	cm
*CCLM_Wd*	Bycatch/Marine turtles	Minimum Curved Carapace Width	cm
*CSLM_Ln*	Bycatch/Marine turtles	Minimum Straight Carapace Length	cm
*CSLM_Wd*	Bycatch/Marine turtles	Straight Carapace Width	cm
*Ttl_ln_*	Bycatch/Dolphins	Total length	cm

The monitoring activity was designed according to fleet dynamics that in the case of midwater pair trawl fishery is highly variable in space - depending on the distribution of the target species (small pelagics) – and time – in terms of the national regulation of fishing effort (i.e., trawlers must respect temporal closures during weekends and spawning periods of the target species) and should operate within Italian waters. Based on these considerations, observations covered between 3 and 7% of the total annual fishing effort of midwater pair trawlers operating in the northern-central Adriatic Sea.

### Database framework

**Fig. 1:**
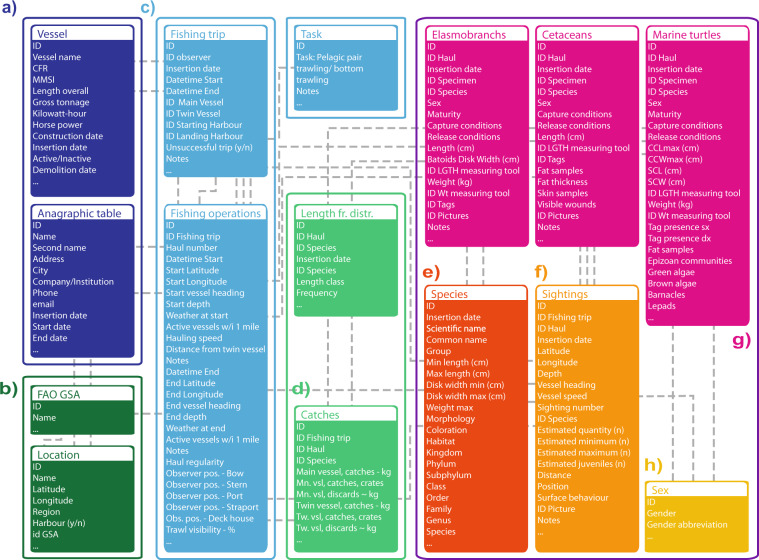
Diagram showing the core database tables. Colours group the information by type: (**a**) anagraphic table and fishing vessels; (**b**) geographical data; (**c**) fishing trips and hauls; (**d**) target species captures and length frequency distributions. The purple box collects biological data of bycatch species, with (**e**) containing data about the species; (**f**) specimens’ sightings; (**g**) represent the core bycatch tables for elasmobranchs, cetaceans and turtles; (**g**) additional data on species and sex is shared by multiple tables. Following the meaning of the codes visible in the Marine turtles table: fr.distr, frequency distribution; CCLmax, Maximum curved carapace length; CCWmax, (cm) Maximum curved carapace width; SCL, Straight carapace length; SCW, Straight carapace width.

**Fig. 2:**
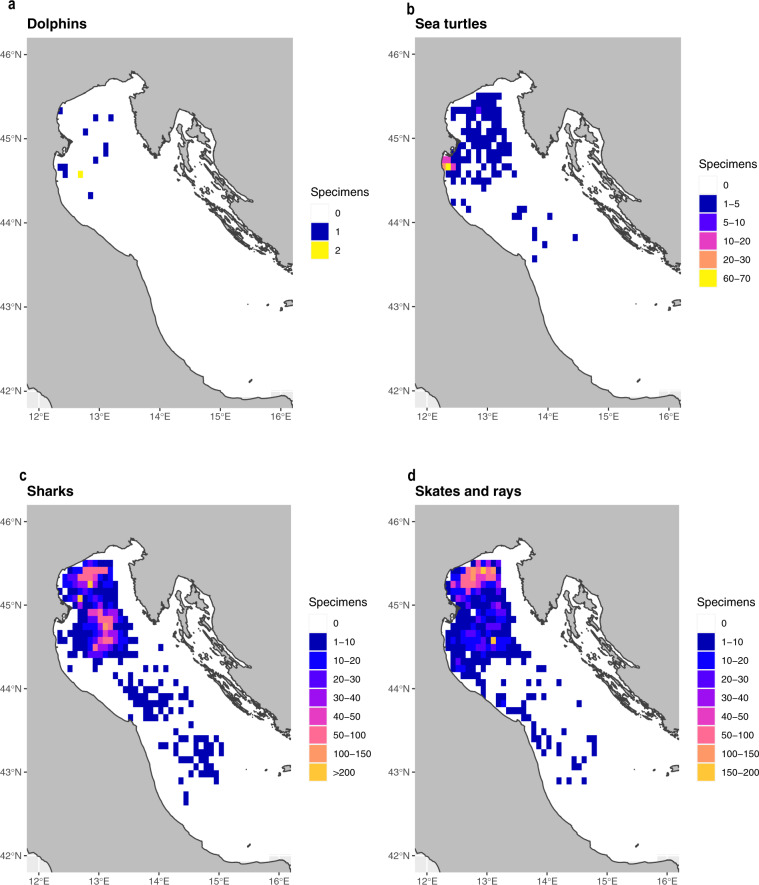
Distribution of total bycatch of (**a**) dolphins; (**b**) sea turtles; (**c**) sharks and (**d**) skates and rays recorded between 2006 and 2019 in the northern central Adriatic Sea. The number of individuals per taxonomic group is represented by a colour scale applied to a grid with 5 nm cells.

**Fig. 3:**
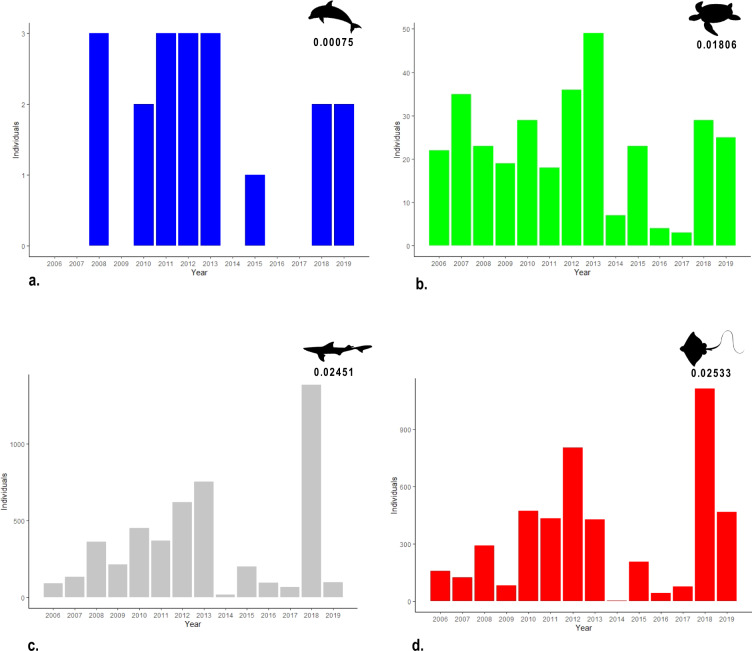
Number of specimens of (**a**) dolphins; (**b**) sea turtles; (**c**) sharks and (**d**) skates and rays recorded between 2006 and 2019 and their relative average estimated bycatch rates (individuals/n. hauls).

All information collected on board by fishery observers were reported in a dedicated spreadsheet. Each file was read and checked for potential erroneous entries by using a series of Python routines. After validation, the data were uploaded in the “BYCATCH” database hosted at the Italian National Research Council (CNR) Institute of Marine Biological Resources and Biotechnologies (IRBIM) of Ancona, Italy. BYCATCH was built in MySQL and it was managed and maintained using Python, R and different database management tools (e.g., phpMyAdmin and MySQL Workbench). BYCATCH consists of a collection of tables that store interrelated data. The main database tables are illustrated in the diagram shown in Fig. 1. Each record is associated to a unique ID which allows to create relationships between tables and to generate different datasets. BYCATCH contains fishing operations of the Italian midwater pair trawl fishery monitored between 2006 and 2019, the geographic distribution and biological information of incidental catches of cetaceans, sea turtles and elasmobranchs and dolphin sightings. An overview of all data stored in BYCATCH is provided Online-only Table 1. The geographic distribution of all bycatch specimens is shown in Fig. 2 and for each species, the number of specimens recorded every year and their bycatch rate are shown in Fig. 3. While BYCATCH was built specifically to house bycatch data of the species noted above, the database structure was designed to easily be applied to other species and to include different type of information (e.g., catches of target species and environmental variables). From BYCATCH, three datasets were extracted via queries directly in MySQL or through the free software environment R^31^ using RMySQL package, a Database Interface and ‘MySQL’ Driver for R^32^.

## Data Records

The three datasets are available on the Marine Data Archive (MDA)^33^. All datasets include data aggregated per year and per cell using grid cells of 5 nm which cover the northern central Adriatic Sea. The datasets consist of a collection of shapefiles arranged in a GeoPackage format^34^. In all shapefiles, the attribute table displays the cell ID and the mean of geographical coordinates of each cell. Then, each shape file contains the following specific information:

(I) Monitored fishing effort of mid-water pair trawlers in the northern central Adriatic Sea. This dataset contains 17654 monitored fishing operations arranged in 14 layers (the time series 2006–2019). Each layer (year) describes the monitored effort in terms of number of hauls per cell (n_Hauls), the number of fishing hours per cell (fsh.hrs), the average haul duration in minutes (avH.Len) per cell, the average towing speed in knots (avHspeed) and the average sea depth in meters (avDepth).
(II) Incidental catches and morphometric data of marine megafauna. This data collection includes 3529 bycatch events arranged in 23 shapefiles (one per species). Three taxonomic groups are represented within the dataset: bottlenose dolphins, loggerhead turtles and elasmobranchs (sharks and rays). Within each shapefiles’ attribute table, species abundance is recorded in terms of number of individuals per cell and per year. The dataset also contains biological and morphological information of 7496 bycatch specimens aggregated by cell. Depending on the taxonomic group, additional information reported by each cell includes morphometric data, namely the average body length (cm), body weight (kg), disc width (cm, only for batoids), carapace measurements for marine turtles, gender classification (females, males, unknown), and the physical conditions of the individual released at sea. An accurate description of all the variables considered is provided in Table 1.
(III) Sightings of bottlenose dolphin (*Tursiops truncatus*). This file contains 6953 observed individuals from 3011 fishing operations aggregated by year and cell.

## Technical Validation

The collection of tables stored in BYCATCH is the result of an intense compilation and validation process of a 14-year time series of marine megafauna bycatch. All information collected on board by fishery observers were reported in a dedicated spreadsheet developed in Microsoft Excel. To preserve the quality of the data, avoiding data entry errors and typing (e.g., wrong entry format, misspelling, missing information), a series of conditional formatting rules were created with Excel Visual Basic for Application (VBA) macro. When the user was filling the spreadsheet, if the data were not validated against a specific entry format or range, the inconsistency was highlighted by an error message. Then, the user could immediately solve any identified issue, and proceed with data entry process. Specifically, the rules were set up to highlight potential:

- Mismatches between fishery observers and their corresponding monitored fishing vessel and harbour of provenience: each fishery observer monitored a specific fishing vessel from a specific harbour.

- Inconsistencies between temporal variables: all fishing operations recorded at specific dates and times should be included within the time frame of the corresponding fishing trip. In addition, the timing of the starting of the hauling should be reported before than the timing of its conclusion.

- Inconsistencies of recorded fishing operations: the duration of each fishing operation recorded on board was compared with an estimated duration, calculated as the ratio between the length (distance between the starting and ending points of a fishing operation), and the average trawling speed (kn) of the vessel. A maximum of 30% of discrepancy between observed and calculated durations was allowed.

- Wrong vessel speed: considering that the usual speed of a midwater pelagic trawler should fall within the 3–5 (maximum) kn speed range, only values inside this range are allowed.

After data validation, all spreadsheets were uploaded in BYCATCH through Python, and tables were updated to hold it in a consistent format. During the upload, the programme repeats some checks on the data (e.g., temporal inconsistency, length and speed of fishing operations) and screen geographical location of fishing operations (e.g., position on land). If an error was found, the wrong record was flagged to be further investigated and corrected.

## Usage Notes

The datasets are freely available and stored in the Marine Data Archive (MDA) and should be appropriately referenced by citing the present paper. The datasets can be used by various end users. For instance, fishery scientists can examine where the Italian midwater pair trawl fishery occurred over 14 years in the northern central Adriatic Sea. Knowing where fishing is occurring is crucial to assess potential impact on different vulnerable species being taken in the basin. From the datasets, the monitored fishing effort can be coupled with Automatic Identification System (AIS) transmitted data to quantify unknown vessel tracks providing a more realistic picture of the overall fishing activity. Then, ecologists can also couple the present historical bycatch data of different species with other existing time series coming from different sources (e.g., monitoring activities from different Mediterranean regions, aerial surveys, commercial landings, interviews with fishers). In this contest, meta-analyses, Bayesian statistics and other approaches can be used to combine and analyse data from a variety of sources to evaluate long-term trends of potential threatened marine megafauna and how their populations have changed over time^14,35–38^. The datasets described in this work can also help conservation biologists and managers to fill gaps in marine megafauna knowledge and to develop more accurately managements strategies.

However, users should take into account some limitations of the datasets. Overall, historical bycatch data of different marine megafauna species exhibit a large number of zero observations (no catch). This is a regular case in fishery, when data regards non-target species, like dolphins, sea turtles and elasmobranchs which are caught less frequently than target species (small pelagic fish in the case of midwater pair trawl fishery). Thus, when evaluating the relative abundance of such species over time, the modelling approach considered should deal with potential interpretation problems^35,36^. Previous studies showed that CPUE data can be modelled to address the effect of operational and environmental factors that can affect catch rate. For instance, historical CPUE of two myliobatids, the common eagle ray (*Myliobatis aquila*) and the bull ray (*Aetomylaeus bovinus*) included in the dataset were modelled by^28^ using Generalized Additive Models (GAMs). This procedure was applied in a delta modelling approach which allowed to model the probability of species occurrence and the magnitude of catch events separately (see details in^28^). The results indicated that the predictive accuracy of the delta-modelling strategy was rather good and a similar approach was used by^30^ to evaluate the seasonal distribution of bycatch events of loggerhead (*Caretta caretta*). Furthermore, Zero-inflated General Linear Models (GLMs) were used by^29^ to examine the relative abundance of four elasmobranch species - common smooth-hound *(Mustelus mustelus*), common eagle ray (*Myliobatis aquila*), spiny dogfish (*Squalus acanthias*), and pelagic stingray (*Pteroplatytrygon violacea*) - included in the dataset. The results aimed to standardize annual trends of the CPUE, considering the best set of covariates among those tested (see details in^29^). In addition, users should consider that the datasets include fishery-dependent data which can suffer of intrinsic bias related to the stochasticity of the distribution of marine megafauna and a lack of a well-defined sampling design of the monitoring activity in space and time. Indeed, following the considerations in^27,29,30^ a non-equal distribution of the monitoring activity was conditioned by fleet dynamics (e.g., fishing closure, fish market preferences and price) and bureaucratic delays of the project, which affected both observed pattern and estimation of bycatch events.

## Supplementary information

Supplementary Information

## Data Availability

The datasets were extracted via queries directly in MySQL Workbench and through the free software environment R^31^, using: the *dbConnect* function from the RMySQL package, a Database Interface and ‘MySQL’ Driver for R^32^. The maps in Fig. 2 were created using several R packages like *tidyverse*^39^ for data handling, *rgdal*^40^ a package providing bindings to the “Geospatial Data Abstraction Library”, *sf* package, a standardized way of encoding spatial vector data in R^41^ and the package *ggplot*^42^ for graphical visualization. The package *geosphere*^43^ was also used for the calculation of haul midpoints, starting from the coordinates of gear deploying and retrieving of each fishing operation. A few examples of these operations (MySQL and R) and of the R code for the creation of the maps are available among the shared datasets attached to this work as Supplementary Information.

## Online-only Table

**Online-only Table 1:** Summary of the data contained in the database BYCATCH (i) fishing effort; (ii) bycatch events; (iii) biological information of all bycatch specimens and (iv) dolphin sightings.

(i) Fishing effort												
Years		Fishing harbour	N. fishing vessel	N. fishing trips	N. hauls	Lat Min and Max		Long Min and Max		Mean haul duration (min)	Mean vessel speed (nm)	Mean depth (m)
2006–2013; 2015–2019		Chioggia	8	1117	5108	43.9341	45.5685	12.3127	13.9967	43.14	4.45	29.63
2007–2019		Pila di Porto Tolle	7	560	2223	44.2883	45.4945	12.3271	13.4592	32.31	4.14	32.75
2019		Goro	1	17	40	44.6273	44.8751	12.3659	12.5954	30.40	3.97	17.39
2006–2019		Porto Garibaldi	11	1566	6064	43.8894	45.0973	12.2505	13.4496	36.05	4.41	27.23
2014; 2016; 2018–2019		Cesenatico	3	154	498	43.9586	44.5523	12.3945	13.0217	45.39	4.25	14.49
2017		Rimini	1	5	19	43.8438	44.5918	12.499	13.2343	38.02	4.56	22.11
2018		Cattolica	1	21	78	43.801	44.4999	12.622	13.356	30.53	4.32	27.96
2009–2013; 2015–2019		Ancona	21	452	1866	42.8754	44.3962	13.0775	14.9715	57.06	4.12	61.78
2009–2019		San Benedetto del Tronto	9	354	1303	42.6176	43.9546	13.7378	15.1347	76.44	4.14	78.34
2006–2010; 2018		Giulianova	6	146	459	42.4998	43.6215	13.9979	15.1178	77.52	4.09	85.30
**(ii) Bycatch events**												
**Years**		**N. Fishing trip**	**N. hauls**	**Lat Min and Max**		**Lon Min and Max**		**Species**			**Events**	
2008–2013; 2015; 2018–2019		3924	15918	44.3243	45.3311	12.3501	13.173	*Tursiops truncatus*			12	
2006–2019		4392	17658	43.568	45.5274	12.2591	14.4655	*Caretta caretta*			254	
2006–2013; 2015–2016; 2018–2019		4128	16616	44.446	45.4802	12.4936	13.4937	*Alopias vulpinus*			35	
2006; 2010; 2019		1044	4028	44.2464	44.6513	12.312	12.5325	*Carcharhinus plumbeus*			3	
2007; 2008; 2014		748	2938	44.5806	45.4116	12.4577	13.2357	*Mustelus asterias*			8	
2006–2013; 2015–2019		4300	17334	43.1347	45.5352	12.3668	14.9923	*Mustelus mustelus*			442	
2007–2010; 2012–2019		3867	15249	42.722	45.534	12.389	15.0125	*Mustelus punctulatus*			121	
2008–2010; 2012–2013; 2016–2019		3136	12412	43.0195	45.3998	12.3868	14.6869	*Prionace glauca*			22	
2007–2011; 2013; 2018		2467	10233	44.4514	45.1344	12.8173	13.3777	*Scyliorhinus canicula*			12	
2018		382	1484	45.0696	45.2912	12.6917	13.1555	*Scyliorhinus stellaris*			3	
2006–2019		4392	17658	42.6312	45.4419	12.3584	15.0194	*Squalus acanthias*			912	
2006–2013; 2015–2019		4300	17334	43.4842	45.5341	12.3872	13.9744	*Aetomylaeus bovinus*			213	
2006–2013; 2015–2016; 2018		3595	14781	43.1101	45.231845.534	12.387	14.614714.811	*Dasyatis pastinaca*			20	
2006–2013; 2015–2019		4300	17334	43.0962	45.5284	12.3271	14.636	*Myliobatis aquila*			800	
2006–2019		4392	17658	42.8805	44.8984	12.3102	13.8564	*Pteroplatytrygon violacea*			569	
2006–2007; 2011–2013		1610	6942	44.2541	44.9816	12.7179	13.9227	*Raja asterias*			11	
2007–2013; 2015–2019		4155	16625	44.2001	44.6138	12.4241	13.253	*Raja clavata*			79	
2013		355	1629	44.6138	44.6977	13.253	13.7111	*Raja miraletus*			1	
2007; 2010; 2012		1260	5049	44.0464		12.6415		*Torpedo marmorata*			5	
**(iii) Biological information of all bycatch specimens**												
**Species**		**N. specimens**	**List of morphometric data**									
*Tursiops truncatus*		13	Body length (m); Sex; Capture/release conditions									
*Caretta caretta*		319	Minimum Curved Carapace Length (cm); Minimum Curved Carapace Width (cm); Minimum Straight Carapace Length (cm); Straight Carapace Width (cm); Weight (Kg); Sex; Capture/release conditions									
*Alopias vulpinus*		38	Body length (cm); Weight (Kg); Sex; Tag ID; Capture/release conditions									
*Carcharhinus plumbeus*		3										
*Mustelus asterias*		18										
*Mustelus mustelus*		991										
*Mustelus punctulatus*		231										
*Prionace glauca*		22										
*Scyliorhinus canicula*		12										
*Scyliorhinus stellaris*		3										
*Squalus acanthias*		2369										
*Aetomylaeus bovinus*		317	Body length (cm); Disc width (cm); Weight (Kg); Sex; Tag ID; Capture/release conditions									
*Dasyatis pastinaca*		51										
*Myliobatis aquila*		2179										
*Pteroplatytrygon violacea*		785										
*Raja asterias*		16										
*Raja clavata*		114										
*Raja miraletus*		1										
*Torpedo marmorata*		5										
**(iv) Dolphin sighting**												
**Years**	**Days**	**Species**	**N. individuals**		**Observer position**					**Distance (m)**		
2007–2019	2997	*Tursiops truncatus*	6953		Bow; Stern; Deck house; Beam					<10; 10–50; 51–100; 101–500; >500		

## References

[1] Lewison RL, Crowder LB, Read AJ, Freeman SA (2004). Understanding impacts of fisheries bycatch on marine megafauna. Trends Ecol. Evol..

[2] Lewison RL (2014). Global patterns of marine mammal, seabird, and sea turtle bycatch reveal taxa-specific and cumulative megafauna hotspots. Proc. Natl. Acad. Sci. USA.

[3] Davies RWD, Cripps SJ, Nickson A, Porter G (2009). Defining and estimating global marine fisheries bycatch. Mar. Policy.

[4] Oliver S, Braccini M, Newman SJ, Harvey ES (2015). Global patterns in the bycatch of sharks and rays. Mar. Policy.

[5] Ortuño Crespo G, Dunn DC (2017). A review of the impacts of fisheries on open-ocean ecosystems. ICES J. Mar. Sci..

[6] Wallace BP, Heppell SS, Lewison RL, Kelez S, Crowder LB (2008). Impacts of fisheries bycatch on loggerhead turtles worldwide inferred from reproductive value analyses. J. Appl. Ecol..

[7] Dulvy NK (2014). Extinction risk and conservation of the world’s sharks and rays. eLife.

[8] Lotze HK, Mills Flemming J, Magera AM (2017). Critical factors for the recovery of marine mammals. Conserv. Biol..

[9] Carpentieri, P., Nastasi, A., Sessa, M. & Srour, A. *Incidental catch of vulnerable species in Mediterranean and Black Sea fisheries – A review*. Studies and Reviews No. 101 (General Fisheries Commission for the Mediterranean). Rome, FAO, 2021.

[10] European Parliament, Council of the European Union. Council Regulation No. 199/2008 of 25 February 2008 concerning the establishment of a Community framework for the collection, management and use of data in the fisheries sector and support for scientific advice regarding the Common Fisheries Policy, OJ L 60/1, 5.3.2008, 1–12 (European Council, 2008).

[11] Savoca MS (2020). Comprehensive bycatch assessment in US fisheries for prioritizing management. Nat. Sustain..

[12] Bearzi, G. *Interactions between cetacean and fisheries in the Mediterranean Sea. Cetaceans of the Mediterranean and Black Seas: state of knowledge and conservation strategies*. (ACCOBAMS Secretariat, Monaco, 2002).

[13] Casale P (2011). Sea turtle by-catch in the Mediterranean. Fish Fish..

[14] Ferretti F, Myers RA, Serena F, Lotze H (2008). Loss of large predatory sharks from the Mediterranean Sea. Conserv. Biol..

[15] Dulvy, N. K., Allen, D. J., Ralph, G. M. & Walls, R. H. L. *The conservation status of sharks, rays and chimaeras in the Mediterranean Sea* (IUCN, Malaga, Spain, 2016).

[16] FAO. *Monitoring the incidental catch of vulnerable species in Mediterranean and Black Sea fisheries: Methodology for data collection*. FAO Fisheries and Aquaculture Technical Paper No. 640. (Rome, FAO, 2019).

[17] Mazzoldi C, Sambo A, Riginella E (2014). The Clodia database: a long time series of fishery data from the Adriatic Sea. Sci. Data.

[18] Fortibuoni T (2017). Fish and fishery historical data since the 19th century in the Adriatic Sea, Mediterranean. Sci. Data.

[19] Mancusi C (2020). MEDLEM database, a data collection on large Elasmobranchs in the Mediterranean and Black seas. Mediterr. Mar. Sci..

[20] Pulcinella, J. *et al*. Institute for Biological Resources and Marine Biotechnologies (IRBIM), Italian National Institute for Environmental Protection and Research (ISPRA). Monitored bycatch of *Mustelus mustelus, Squalus acanthias, Myliobatis aquila, Pteroplatytrygon violacea* and *Caretta caretta* in mid-water pair trawl fishery in the northern Adriatic Sea, from 2006-2017 (2020).

[21] Cardinale M, Osio GC, Scarcella G (2017). Mediterranean Sea: a failure of the European fisheries management system. Front. Mar. Sci.

[22] Lotze HK, Coll M, Dunne J (2011). Historical changes in marine resources, food-web structure and ecosystem functioning in the Adriatic Sea. Ecosystems.

[23] Giani M (2012). Recent changes in the marine ecosystems of the northern Adriatic Sea. Estuar. Coast. Shelf Sci..

[24] Romano B, Zullo F (2014). The urban transformation of Italy’s Adriatic coastal strip: Fifty years of unsustainability. Land Use Policy.

[25] Holcer, D. & Fortuna, C.M. *Status and conservation of cetaceans the Adriatic Sea*. (UNEP-MAP-RAC-SPA. Malaga, 2014).

[26] Ferretti, M. *Technological evolution of pelagic trawl fishery in the Adriatic, Italy*. (FAO Fisheries Reports FAO, 1981).

[27] Fortuna CM (2010). Bycatch of cetaceans and other species of conservation concern during pair trawl fishing operations in the Adriatic Sea (Italy). Chem. Ecol..

[28] La Mesa G, Annunziatellis A, Filidei E, Fortuna CM (2016). Bycatch of myliobatid rays in the central Mediterranean Sea: the influence of spatiotemporal, environmental, and operational factors as determined by generalized additive modelling. Mar. Coast. Fish.

[29] Bonanomi S, Pulcinella J, Fortuna CM, Moro F, Sala A (2018). Elasmobranch bycatch in the Italian Adriatic pelagic trawl fishery. PLoS One.

[30] Pulcinella J (2019). Bycatch of loggerhead turtle (*Caretta caretta*) in the Italian Adriatic midwater pair trawl fishery. Front. Mar. Sci..

[31] R Core Team R: A Language and Environment for Statistical Computing. R Foundation for Statistical Computing, Vienna, Austria. https://www.R-project.org/ (2016).

[32] James, D. A., DebRoy, S. & Horner, M. J. Package ‘RMySQL’. R documentation. http://cran.rproject.org/web/packages/RMySQL/RMySQL.Pdf (2012).

[33] Bonanomi S (2021). Marine Data Archive.

[34] Open Geospatial Consortium OGC, 2017. GeoPackage Implementations [WWW Document]. OGC implementations. URL https://www.GeoPackage.org/implementations.html.

[35] Ferretti, F., Crowder, L. B. & Micheli, F. *Marine Historical Ecology in Conservation: Applying the Past to Manage for the Future Vol*. (ed. Kittinger, J. N., McClenachan, L., Gedan, K. B. & Blight L. K.) Ch. 4 (California Univ. Press, 2014).

[36] Brodziak J, Walsh WA (2013). Model selection and multimodel inference for standardizing catch rates of bycatch species: a case study of oceanic whitetip shark in the Hawaii-based longline fishery. *Can*. J. Fish. Aquat. Sci..

[37] Orio A (2017). Modelling indices of abundance and size-based indicators of cod and flounder stocks in the Baltic Sea using newly standardized trawl survey data. ICES J. Mar. Sci..

[38] McClenachan L, Ferretti F, Baum JK (2012). From archives to conservation: why historical data are needed to set baselines for marine animals and ecosystems. Conserv. Lett..

[39] Wickham, H. The tidyverse. R package ver, 1(1), 1 (2017).

[40] Bivand, R. *et al*. rgdal: bindings for the geospatial data abstraction library 2017. R package version 0.8-13 (2018).

[41] Pebesma EJ (2018). Simple features for R: Standardized support for spatial vector data. R J..

[42] Wickham, H. ggplot2: Elegant graphics for data analysis (2nd ed.). New York, NY: Springer (2016).

[43] Hijmans, R. J., Williams, E. & Vennes, C. The” geosphere” package (Version 1.2. 1) 2010.

