# Supplementary material for: A 14-year time series of marine megafauna bycatch in the Italian midwater pair trawl fishery
Source: Sci Data. 2022 Feb 14;9:51. doi: 10.1038/s41597-022-01155-2 (PMC8844044; doi:10.1038/s41597-022-01155-2)
Supplement: Supplementary file 1 — Supplementary Information [file 41597_2022_1155_MOESM1_ESM.pdf]

```sql
use bycatch;
# Example of captured specimens count by haul and species
SELECT id_cala
, id_specie, count(id_specie)
FROM elasmobranchi_cattura ec
GROUP BY id_cala, id_specie
ORDER BY id_cala asc
LIMIT 0, 20000;

# Turtles
SELECT
op.id_uscita as id_uscita,
op.id as id_cala,
g.name as gsa,
l.nome as harbour,
v.nome as Vessel,
Date(datetime_cala) as Date, YEAR(datetime_cala) as Year,
MONTH(datetime_cala) as month, TIME(datetime_cala) as Time,
op.COG_Cala as route,
op.Lat_Cala, op.Lon_Cala,
op.Lat_Salpa, op.Lon_Salpa,
op.Velocita_Traina_nm_h as speed, op.Prof_Cala_m as depth,
TIMEDIFF(TIME(op.datetime_salpa ), TIME(op.datetime_cala)) as duration,
b.scala as beaufort_scale, b.velocita_vento_nds as velocita_vento_nds,
op.mitigazione, # da vedere con Sara
sp.Nome_scientifico as specie, count(tc.ID_Specie) as Specimens
FROM tartarughe_cattura tc
LEFT JOIN specie sp ON tc.ID_Specie = sp.id
LEFT JOIN operazioni_pesca op ON tc.id_cala = op.id
LEFT JOIN uscite u ON op.id_uscita = u.id
LEFT JOIN localita l on u.Id_porto_partenza = l.id
LEFT JOIN task on u.id_task = task.id
LEFT JOIN vessel v ON u.id_vessel_principale = v.id
LEFT JOIN gsa g ON op.gsa_salpa = g.id
LEFT JOIN beaufort b ON op.id_beaufort_cala = b.id
WHERE u.id_task = 1 AND g.name != 'GSA16'
GROUP BY tc.id_cala, tc.id_specie
ORDER BY  Date(datetime_cala)
LIMIT 0, 20000;



# Elasmobranchs
#Select * FROM elasmobranchi_cattura;
SELECT
op.id_uscita as id_uscita,
op.id as id_cala,
g.name as gsa,
l.nome as harbour,
v.nome as Vessel,
Date(datetime_cala) as Date, YEAR(datetime_cala) as Year,
MONTH(datetime_cala) as month, TIME(datetime_cala) as Time,
op.COG_Cala as route,
op.Lat_Cala, op.Lon_Cala,
op.Lat_Salpa, op.Lon_Salpa,
op.Velocita_Traina_nm_h as speed, op.Prof_Cala_m as depth,
TIMEDIFF(TIME(op.datetime_salpa ), TIME(op.datetime_cala)) as duration,
b.scala as beaufort_scale, b.velocita_vento_nds as velocita_vento_nds,
op.mitigazione, # da vedere con Sara
sp.Nome_scientifico as specie, count(ec.id_specie) as Specimens
FROM elasmobranchi_cattura ec
LEFT JOIN specie sp ON ec.id_specie = sp.id
LEFT JOIN operazioni_pesca op ON ec.id_cala = op.id
LEFT JOIN uscite u ON op.id_uscita = u.id
LEFT JOIN localita l on u.Id_porto_partenza = l.id
LEFT JOIN task on u.id_task = task.id
LEFT JOIN vessel v ON u.id_vessel_principale = v.id
```

```sql
LEFT JOIN gsa g ON op.gsa_salpa = g.id
LEFT JOIN beaufort b ON op.id_beaufort_cala = b.id
WHERE u.id_task = 1
GROUP BY ec.id_cala, ec.id_specie
ORDER BY  Date(datetime_cala)
LIMIT 0, 20000;

# Dolphins
Select * FROM cetacei_cattura
ORDER BY id_cala;
SELECT
op.id_uscita as id_uscita,
op.id as id_cala,
g.name as gsa,
l.nome as harbour,
v.nome as Vessel,
Date(datetime_cala) as Date, YEAR(datetime_cala) as Year,
MONTH(datetime_cala) as month, TIME(datetime_cala) as Time,
op.COG_Cala as route,
op.Lat_Cala, op.Lon_Cala,
op.Lat_Salpa, op.Lon_Salpa,
op.Velocita_Traina_nm_h as speed, op.Prof_Cala_m as depth,
TIMEDIFF(TIME(op.datetime_salpa ), TIME(op.datetime_cala)) as duration,
b.scala as beaufort_scale, b.velocita_vento_nds as velocita_vento_nds,
op.mitigazione, # da vedere con Sara
sp.Nome_scientifico as specie, count(cc.id_specie) as Specimens
FROM cetacei_cattura cc
LEFT JOIN specie sp ON cc.id_specie = sp.id
LEFT JOIN operazioni_pesca op ON cc.id_cala = op.id
LEFT JOIN uscite u ON op.id_uscita = u.id
LEFT JOIN localita l on u.Id_porto_partenza = l.id
LEFT JOIN task on u.id_task = task.id
LEFT JOIN vessel v ON u.id_vessel_principale = v.id
LEFT JOIN gsa g ON op.gsa_salpa = g.id
LEFT JOIN beaufort b ON op.id_beaufort_cala = b.id
WHERE g.name != "GSA16" and t.nome  != 'Task5.1b' and l.nome !="Sciacca"
AND check_catch_real !=0
GROUP BY cc.id_cala, cc.id_specie
ORDER BY  Date(datetime_cala)
LIMIT 0, 20000;
##########################################################################################
######## Aggregated by year ##############################################################
############## solo gsa 17  o NULL #######################################################
#Turtles
SELECT Year, count(id_uscita) as Events,
specie,
sum(Specimens) as Specimens
FROM(
# Tartarughe
SELECT
op.id_uscita as id_uscita,
op.id as id_cala,
g.name as gsa,
l.nome as harbour,
v.nome as Vessel,
Date(datetime_cala) as Date, YEAR(datetime_cala) as Year,
MONTH(datetime_cala) as month, TIME(datetime_cala) as Time,
op.COG_Cala as route,
op.Lat_Cala, op.Lon_Cala,
op.Lat_Salpa, op.Lon_Salpa,
op.Velocita_Traina_nm_h as speed, op.Prof_Cala_m as depth,
TIMEDIFF(TIME(op.datetime_salpa ), TIME(op.datetime_cala)) as duration,
b.scala as beaufort_scale, b.velocita_vento_nds as velocita_vento_nds,
op.mitigazione, # da vedere con Sara
sp.Nome_scientifico as specie, count(tc.ID_Specie) as Specimens
FROM tartarughe_cattura tc
LEFT JOIN specie sp ON tc.ID_Specie = sp.id
```

```
LEFT JOIN operazioni_pesca op ON tc.id_cala = op.id
LEFT JOIN uscite u ON op.id_uscita = u.id
LEFT JOIN localita l on u.Id_porto_partenza = l.id
LEFT JOIN task on u.id_task = task.id
LEFT JOIN vessel v ON u.id_vessel_principale = v.id
LEFT JOIN gsa g ON op.gsa_salpa = g.id
LEFT JOIN beaufort b ON op.id_beaufort_cala = b.id
WHERE u.id_task = 1 and g.name!= "GSA16"
GROUP BY tc.id_cala, tc.id_specie
ORDER BY  Date(datetime_cala)
LIMIT 0, 20000
)as q1
GROUP BY Year, specie
LIMIT 0, 20000;



#Elasmobranchs
SELECT Year, count(id_uscita) as Events,
specie,
sum(Specimens) as Specimens
FROM(
SELECT
op.id_uscita as id_uscita,
op.id as id_cala,
g.name as gsa,
l.nome as harbour,
v.nome as Vessel,
Date(datetime_cala) as Date, YEAR(datetime_cala) as Year,
MONTH(datetime_cala) as month, TIME(datetime_cala) as Time,
op.COG_Cala as route,
op.Lat_Cala, op.Lon_Cala,
op.Lat_Salpa, op.Lon_Salpa,
op.Velocita_Traina_nm_h as speed, op.Prof_Cala_m as depth,
TIMEDIFF(TIME(op.datetime_salpa ), TIME(op.datetime_cala)) as duration,
b.scala as beaufort_scale, b.velocita_vento_nds as velocita_vento_nds,
op.mitigazione, # da vedere con Sara
sp.Nome_scientifico as specie, count(ec.id_specie) as Specimens
FROM elasmobranchi_cattura ec
LEFT JOIN specie sp ON ec.id_specie = sp.id
LEFT JOIN operazioni_pesca op ON ec.id_cala = op.id
LEFT JOIN uscite u ON op.id_uscita = u.id
LEFT JOIN localita l on u.Id_porto_partenza = l.id
LEFT JOIN task on u.id_task = task.id
LEFT JOIN vessel v ON u.id_vessel_principale = v.id
LEFT JOIN gsa g ON op.gsa_salpa = g.id
LEFT JOIN beaufort b ON op.id_beaufort_cala = b.id
WHERE u.id_task = 1 and g.name!= "GSA16"
GROUP BY ec.id_cala, ec.id_specie
ORDER BY  Date(datetime_cala)
LIMIT 0, 20000) as q1
GROUP BY Year, specie
LIMIT 0, 20000;


# Dolphins

SELECT Year, count(id_uscita) as Events,
specie,
sum(Specimens) as Specimens
FROM(SELECT
op.id_uscita as id_uscita,
op.id as id_cala,
g.name as gsa,
l.nome as harbour,
v.nome as Vessel,
Date(datetime_cala) as Date, YEAR(datetime_cala) as Year,
```

```
MONTH(datetime_cala) as month, TIME(datetime_cala) as Time,
op.COG_Cala as route,
op.Lat_Cala, op.Lon_Cala,
op.Lat_Salpa, op.Lon_Salpa,
op.Velocita_Traina_nm_h as speed, op.Prof_Cala_m as depth,
TIMEDIFF(TIME(op.datetime_salpa ), TIME(op.datetime_cala)) as duration,
b.scala as beaufort_scale, b.velocita_vento_nds as velocita_vento_nds,
op.mitigazione, # da vedere con Sara
sp.Nome_scientifico as specie, count(cc.id_specie) as Specimens
FROM cetacei_cattura cc
LEFT JOIN specie sp ON cc.id_specie = sp.id
LEFT JOIN operazioni_pesca op ON cc.id_cala = op.id
LEFT JOIN uscite u ON op.id_uscita = u.id
LEFT JOIN localita l on u.Id_porto_partenza = l.id
LEFT JOIN task on u.id_task = task.id
LEFT JOIN vessel v ON u.id_vessel_principale = v.id
LEFT JOIN gsa g ON op.gsa_salpa = g.id
LEFT JOIN beaufort b ON op.id_beaufort_cala = b.id
WHERE g.name != "GSA16" and t.nome  != 'Task5.1b' and l.nome !="Sciacca"
AND check_catch_real !=0
GROUP BY cc.id_cala, cc.id_specie
ORDER BY  Date(datetime_cala)
LIMIT 0, 20000
)
 as q1
GROUP BY Year, specie
LIMIT 0, 20000;
```

```
use bycatch;
SELECT count(*) FROM cetacei_cattura; # sono 23, 2008-2019
SELECT count(*) FROM elasmobranchi_cattura; #8546 records
SELECT count(*) FROM tartarughe_cattura; #341

# Dolphins
SELECT * FROM cetacei_cattura ORDER BY datetime;
SELECT
l.nome as harbour,
YEAR(datetime) as Year,
MONTH(datetime) as Month,
sp.Nome_Scientifico,
#FishingTrips
#FishingHauls
#Duration
COUNT(cc.id) as BycatchEvents,
SUM(cc.n_tot_catture) as Individuals
#BycatchRate
#BPUE
FROM cetacei_cattura cc
LEFT JOIN specie sp ON cc.id_specie = sp.id
LEFT JOIN operazioni_pesca op ON cc.id_cala = op.id
LEFT JOIN uscite u ON op.id_uscita = u.id
LEFT JOIN localita l on u.Id_porto_partenza = l.id
GROUP BY harbour, Year, Month, Nome_Scientifico
ORDER BY Year, MONTH;



#Fishing trips
SELECT datetime_partenza,
l.nome as harbour,
YEAR(u.datetime_partenza) as YEAR
FROM uscite u
LEFT JOIN localita l on u.Id_porto_partenza = l.id
WHERE u.id_task = 1# Task 5.1a
AND Id_porto_partenza IS NOT NULL
ORDER BY YEAR ASC
LIMIT 0, 20000;


#Task
SELECT DISTINCT(id_task), t.nome FROM uscite
LEFT JOIN task t ON uscite.id_task = t.id;

# Number of fishing trips and hauls per harbour, year and month
SELECT
CONCAT(l.nome, YEAR(u.datetime_partenza), MONTH(u.datetime_partenza)  ) as id,
l.nome as harbour,
YEAR(u.datetime_partenza) as YEAR,
MONTH(u.datetime_partenza) as Month,
COUNT(DISTINCT(u.id)) as FishingTrips,
COUNT(op.id) as FishingHauls,
SUM(time_to_sec(TIMEDIFF(datetime_salpa, datetime_cala)))/3600 as Duration
FROM operazioni_pesca op
LEFT JOIN uscite u ON op.id_uscita = u.id
LEFT JOIN localita l on u.Id_porto_partenza = l.id
WHERE u.id_task = 1# Task 5.1a
GROUP BY YEAR, MONTH, harbour
ORDER BY YEAR
LIMIT 0, 20000;

###################### All together: Dolphins ############################

SELECT
  C.harbour, C.Year, C.Month, C.Nome_Scientifico, C.BycatchEvents, Individuals, #from C
  FishingTrips, FishingHauls, Duration,
```

```
# calcoli
BycatchEvents/FishingHauls AS BycatchRate,
Individuals/Duration AS BPUE
  FROM
(SELECT
#id
concat(harbour, Year, Month) as id,
#tutto il resto
harbour, Year, Month, Nome_Scientifico,
count(id_cala) AS BycatchEvents, sum(Individuals) as Individuals FROM
(SELECT
l.nome as harbour, u.id_task,
YEAR(datetime_cala) as Year,
MONTH(datetime_cala) as Month,
cc.id_cala, s.Nome_Scientifico,
COUNT(cc.id_cala) as Individuals
 FROM cetacei_cattura cc
 LEFT JOIN specie sp ON cc.id_specie = sp.id
 LEFT JOIN operazioni_pesca op ON cc.id_cala = op.id
 LEFT JOIN uscite u ON op.id_uscita = u.id
 LEFT JOIN specie s ON cc.id_specie= s.id
 LEFT JOIN localita l on u.Id_porto_partenza = l.id
 LEFT JOIN task on u.id_task = task.id
 WHERE u.id_task = 1
 GROUP BY id_cala, s.Nome_Scientifico
 ORDER BY YEAR(datetime_cala), MONTH(datetime_cala),
 l.nome, s.Nome_Scientifico) as A
 GROUP BY YEAR, Month, harbour, Nome_Scientifico
 LIMIT 0, 20000) AS C

LEFT JOIN

(SELECT
CONCAT(l.nome, YEAR(u.datetime_partenza), MONTH(u.datetime_partenza)  ) as id,
l.nome as harbour,
YEAR(u.datetime_partenza) as YEAR,
MONTH(u.datetime_partenza) as Month,
COUNT(DISTINCT(u.id)) as FishingTrips,
COUNT(op.id) as FishingHauls,
SUM(time_to_sec(TIMEDIFF(datetime_salpa, datetime_cala)))/3600 as Duration
FROM operazioni_pesca op
LEFT JOIN uscite u ON op.id_uscita = u.id
LEFT JOIN localita l on u.Id_porto_partenza = l.id
WHERE u.id_task = 1# Task 5.1a
GROUP BY YEAR, MONTH, harbour
ORDER BY YEAR
LIMIT 0, 50000) AS D
ON C.id = D.id
LIMIT 0, 50000;




# Turtles
SELECT
  C.harbour, C.Year, C.Month, C.Nome_Scientifico, C.BycatchEvents, Individuals, #from C
  FishingTrips, FishingHauls, Duration,
# calcoli
BycatchEvents/FishingHauls AS BycatchRate,
Individuals/Duration AS BPUE
  FROM
 (SELECT
 ### id ###
 CONCAT(harbour, Year, Month) as id,
 ### tutto il resto ###
 harbour, Year, Month, Nome_Scientifico,
count(id_cala) AS BycatchEvents, sum(Individuals) as Individuals FROM
(SELECT
```

```
l.nome as harbour, u.id_task,
YEAR(u.datetime_partenza) as Year,
MONTH(u.datetime_partenza) as Month,
tc.id_cala, s.Nome_Scientifico,
COUNT(tc.id_cala) as Individuals
 FROM tartarughe_cattura tc
 LEFT JOIN specie sp ON tc.id_specie = sp.id
 LEFT JOIN operazioni_pesca op ON tc.id_cala = op.id
 LEFT JOIN uscite u ON op.id_uscita = u.id
 LEFT JOIN specie s ON tc.id_specie= s.id
 LEFT JOIN localita l on u.Id_porto_partenza = l.id
 LEFT JOIN task on u.id_task = task.id
 WHERE u.id_task = 1# Task 5.1a
 GROUP BY id_cala, Nome_Scientifico
 ORDER BY YEAR(u.datetime_partenza), MONTH(u.datetime_partenza),
 l.nome, s.Nome_Scientifico) as A
 GROUP BY YEAR, Month, harbour, Nome_Scientifico
 LIMIT 0, 20000) AS C

 LEFT JOIN

(SELECT
CONCAT(l.nome, YEAR(u.datetime_partenza), MONTH(u.datetime_partenza)  ) as id,
l.nome as harbour,
YEAR(u.datetime_partenza) as YEAR,
MONTH(u.datetime_partenza) as Month,
COUNT(DISTINCT(u.id)) as FishingTrips,
COUNT(op.id) as FishingHauls,
SUM(time_to_sec(TIMEDIFF(datetime_salpa, datetime_cala)))/3600 as Duration
FROM operazioni_pesca op
LEFT JOIN uscite u ON op.id_uscita = u.id
LEFT JOIN localita l on u.Id_porto_partenza = l.id
WHERE u.id_task = 1# Task 5.1a
GROUP BY YEAR, MONTH, harbour
ORDER BY YEAR
LIMIT 0, 50000) AS D
ON C.id = D.id
LIMIT 0, 50000;

# Elasmobranchs
SELECT
  C.harbour, C.Year, C.Month, C.Nome_Scientifico, C.BycatchEvents, Individuals, #from C
  FishingTrips, FishingHauls, Duration,
# calcoli
BycatchEvents/FishingHauls AS BycatchRate,
Individuals/Duration AS BPUE
  FROM
 (SELECT
 ### id ###
 CONCAT(harbour, Year, Month) as id,
 ### tutto il resto ###
 harbour, Year, Month, Nome_Scientifico,
count(id_cala) AS BycatchEvents, sum(Individuals) as Individuals FROM
(SELECT
l.nome as harbour, u.id_task,
YEAR(u.datetime_partenza) as Year,
MONTH(u.datetime_partenza) as Month,
ec.id_cala, s.Nome_Scientifico,
COUNT(ec.id_cala) as Individuals
 FROM elasmobranchi_cattura ec
 LEFT JOIN specie sp ON ec.id_specie = sp.id
 LEFT JOIN operazioni_pesca op ON ec.id_cala = op.id
 LEFT JOIN uscite u ON op.id_uscita = u.id
 LEFT JOIN specie s ON ec.id_specie= s.id
 LEFT JOIN localita l on u.Id_porto_partenza = l.id
 LEFT JOIN task on u.id_task = task.id
 WHERE u.id_task = 1
```

```
 GROUP BY id_cala,  Nome_Scientifico
 ORDER BY YEAR(u.datetime_partenza), MONTH(u.datetime_partenza),
 l.nome, s.Nome_Scientifico) as A
 GROUP BY YEAR, Month, harbour, Nome_Scientifico
 LIMIT 0, 20000) AS C

 LEFT JOIN

(SELECT
CONCAT(l.nome, YEAR(u.datetime_partenza), MONTH(u.datetime_partenza)  ) as id,
l.nome as harbour,
YEAR(u.datetime_partenza) as YEAR,
MONTH(u.datetime_partenza) as Month,
COUNT(DISTINCT(u.id)) as FishingTrips,
COUNT(op.id) as FishingHauls,
SUM(time_to_sec(TIMEDIFF(datetime_salpa, datetime_cala)))/3600 as Duration
FROM operazioni_pesca op
LEFT JOIN uscite u ON op.id_uscita = u.id
LEFT JOIN localita l on u.Id_porto_partenza = l.id
WHERE u.id_task = 1# Task 5.1a
GROUP BY YEAR, MONTH, harbour
ORDER BY YEAR
LIMIT 0, 50000) AS D
ON C.id = D.id
LIMIT 0, 50000;

########### Accuracy Test -  Observe individuals by port, year and month in a random Harbour/species
combination #####

SELECT
l.nome as harbour, u.id_task,
YEAR(u.datetime_partenza) as Year,
MONTH(u.datetime_partenza) as Month,
s.Nome_Scientifico, ec.id_animale as Identificativo,
ec.id_cala as Cala
#count(ec.id_cala) as Individui ####################
 FROM elasmobranchi_cattura ec
 LEFT JOIN specie sp ON ec.id_specie = sp.id
 LEFT JOIN operazioni_pesca op ON ec.id_cala = op.id
 LEFT JOIN uscite u ON op.id_uscita = u.id
 LEFT JOIN specie s ON ec.id_specie= s.id
 LEFT JOIN localita l on u.Id_porto_partenza = l.id
 LEFT JOIN task on u.id_task = task.id
 WHERE u.id_task = 1 AND
 l.nome = "Chioggia" AND
 s.Nome_Scientifico = 'Mustelus punctulatus' AND
YEAR(u.datetime_partenza) = 2018 AND
MONTH(u.datetime_partenza) = 5
#GROUP BY ec.id_cala ###################
 ORDER BY YEAR(u.datetime_partenza), MONTH(u.datetime_partenza)

 LIMIT 0, 3000;

 SELECT * FROM elasmobranchi_cattura;
```

```
#Morphometric data tables
# Remember "Task"
use bycatch;

SELECT * FROM tartarughe_cattura;
# Tartarughe
SELECT tc.id_cala as id_cala,
s.Nome_Scientifico as Specie,
g.name as GSA,
l.nome as harbour,
cc.nome as Condizioni_Cattura,
gb.nome as Rilascio,
cr.nome as Condizioni_Rilascio,
CCLM_Lungh,
CCLM_Largh,
CSLM_Lungh,
CSLM_Largh,
Peso_KG,
cs.nome_abbr as  Sesso
FROM tartarughe_cattura tc
LEFT JOIN operazioni_pesca op ON tc.id_cala = op.id
LEFT JOIN gsa g  ON op.gsa_salpa = g.id
LEFT JOIN uscite u ON op.id_uscita = u.id
LEFT JOIN localita l on u.Id_porto_partenza = l.id
LEFT JOIN task t ON u.id_task = t.id
LEFT JOIN specie s ON tc.ID_Specie = s.id
LEFT JOIN cattura_condizioni cc ON tc.id_Condizioni_Cattura = cc.id
LEFT JOIN gen_bool gb ON tc.Rilascio = gb.id
LEFT JOIN tartarughe_rilascio tr ON tc.id = tr.id_cattura
LEFT JOIN cattura_condizioni cr ON tr.id_condizioni = cr.id
#LEFT JOIN cattura_rilascio cr ON tc.Rilascio = cr.id
LEFT JOIN cattura_sex cs ON tc.ID_Sesso = cs.id
WHERE g.name != "GSA16" and t.nome  != 'Task5.1b' and l.nome !="Sciacca"
LIMIT 0, 50000;



# Elasmobranchs
SELECT * FROM elasmobranchi_cattura;
SELECT
op.id_uscita as id_uscita,
op.id as id_cala,
g.name as gsa,
l.nome as harbour,
v.nome as Vessel,
Date(datetime_cala) as Date, YEAR(datetime_cala) as Year,
MONTH(datetime_cala) as month, TIME(datetime_cala) as Time,
op.COG_Cala as route,
op.Lat_Cala, op.Lon_Cala,
op.Lat_Salpa, op.Lon_Salpa,
op.Velocita_Traina_nm_h as speed, op.Prof_Cala_m as depth,
timestampdiff(minute,  op.datetime_cala, op.datetime_salpa) as duration,
b.scala as beaufort_scale, b.velocita_vento_nds as velocita_vento_nds,
op.mitigazione, # da vedere con Sara
sp.Nome_scientifico as specie,
cs.nome as sesso,
mat.livello as maturità,
Squali_Lunghezza_cm,
Razze_DW_cm,
peso_kg,
#count(ec.id_specie) as Specimens #non li contiamo più per specie e per cala
id_gen,
id_iso1,
id_iso2,
id_tag,
cc.nome as Condizioni_Cattura,
cr.nome as Rilascio
```

```
FROM elasmobranchi_cattura ec
LEFT JOIN specie sp ON ec.id_specie = sp.id
LEFT JOIN operazioni_pesca op ON ec.id_cala = op.id
LEFT JOIN uscite u ON op.id_uscita = u.id
LEFT JOIN localita l on u.Id_porto_partenza = l.id
LEFT JOIN task on u.id_task = task.id
LEFT JOIN vessel v ON u.id_vessel_principale = v.id
LEFT JOIN gsa g ON op.gsa_salpa = g.id
LEFT JOIN beaufort b ON op.id_beaufort_cala = b.id
LEFT JOIN cattura_sex cs ON ec.Id_Sesso = cs.id
LEFT JOIN maturita mat ON ec.id_maturita = mat.id
LEFT JOIN cattura_condizioni cc ON ec.id_stato_cattura = cc.id
LEFT JOIN cattura_condizioni cr ON ec.id_stato_rilascio = cr.id
WHERE u.id_task = 1  and g.name != "GSA16"  and l.nome !="Sciacca"
#GROUP BY ec.id_cala, ec.id_specie
ORDER BY  Date(datetime_cala)
LIMIT 0, 20000;


# Dolphins
use bycatch;
SELECT * FROM cetacei_cattura;

SELECT op.id_uscita as id_uscita,
op.id as id_cala,
g.name as gsa,
l.nome as harbour,
Date(datetime_cala) as Date, YEAR(datetime_cala) as Year,
s.Nome_Scientifico as Specie,
cs.nome_abbr as  Sesso,
cc.nome as Condizioni_Cattura,
cr.nome as Rilascio,
lungh_tot_cm
FROM cetacei_cattura tc
LEFT JOIN operazioni_pesca op ON tc.id_cala = op.id
LEFT JOIN gsa g  ON op.gsa_salpa = g.id
LEFT JOIN uscite u ON op.id_uscita = u.id
LEFT JOIN localita l on u.Id_porto_partenza = l.id
LEFT JOIN task t ON u.id_task = t.id
LEFT JOIN specie s ON tc.ID_Specie = s.id
LEFT JOIN cattura_condizioni cc ON tc.id_condizione_cattura = cc.id
LEFT JOIN cattura_condizioni cr ON tc.id_condizione_rilascio = cr.id
LEFT JOIN cattura_sex cs ON tc.id_sesso = cs.id
WHERE g.name != "GSA16" and t.nome  != 'Task5.1b' and l.nome !="Sciacca"
AND check_catch_real !=0
LIMIT 0, 50000;








# Dolphin sightings ####


SELECT
op.id_uscita as id_uscita,
av.id_cala as haul,
DATE(op.datetime_cala) as date,
YEAR(op.datetime_cala) as Year,
op.COG_Cala as route,
op.Lat_Cala, op.Lon_Cala,
op.Lat_Salpa, op.Lon_Salpa,
s.Nome_Scientifico as species,
```

```
av.N_quantita as Specimens,
op.Pos_Oss_alla_Salpa_Prua,
op.Pos_Oss_alla_Salpa_Traverso,
op.Pos_Oss_alla_Salpa_Poppa,
op.Pos_Oss_alla_Salpa_Tuga,
d.distanza_m as distance,
gcs.nome as behaviour
FROM avvistamenti av
# informazioni di cala, uscita, porto, gsa
LEFT JOIN operazioni_pesca op ON av.id_cala = op.id
LEFT JOIN uscite u ON op.id_uscita = u.id
LEFT JOIN gsa g ON op.gsa_salpa = g.id
LEFT JOIN localita l on u.Id_porto_partenza = l.id
LEFT JOIN task t on u.id_task = t.id
# altre info
LEFT JOIN specie s ON  av.id_specie =s.id
LEFT JOIN distanze d ON av.id_distanza = d.id
LEFT JOIN gen_comport_di_superficie gcs ON av.comport_di_superficie= gcs.id
WHERE g.name != "GSA16" and t.nome  != 'Task5.1b' and l.nome !="Sciacca"
ORDER BY  Date(op.datetime_cala)
LIMIT 0, 20000;
```

```r
################# Example of data extraction and maps creation ####
# Load packages ####
library("tidyverse")
library("rfishbase")
library("RMySQL")
library("rgdal")
library("rgeos")
library("geosphere")
library("raster")
library("reshape2")
library("ggplot2")
library("lubridate")
library("gridExtra")
library("sp")
library("sf")
library("viridis")
library("RColorBrewer")

# Dataset extraction ####
sql_fun = function(query){
  conn <- dbConnect(MySQL(),
  user="xxxxxxxxxx",
  password="xxxxxxxxxx",
  dbname="bycatch",
  host="xxxxxxxxxx")
  df = dbGetQuery(conn, query)
  # dbDisconnect(conn)
  lapply(dbListConnections(MySQL()), dbDisconnect)
  df
}


# Fishing effort
q_hauls ="SELECT
t.nome as task,
u.id as id_uscita,
op.id as id_cala,
YEAR(datetime_cala) as Year,
DATE(datetime_cala) as date,
g.name as GSA,
l.nome as harbour,
v.nome as vessel,
Lat_Cala, Lon_Cala,
Lat_salpa, Lon_salpa,
(Prof_Cala_m + Prof_Salpa_m)/2 as mean_depth,
 Velocita_Traina_nm_h as speed,
TIME(op.datetime_cala)  as datetime_cala,
TIME(op.datetime_salpa ) as datetime_salpa
 FROM operazioni_pesca op
 LEFT JOIN uscite u
 ON op.id_uscita = u.id
 LEFT JOIN gsa g
 ON op.gsa_salpa = g.id
 LEFT JOIN vessel v
 ON u.id_vessel_principale = v.id
 LEFT JOIN localita l ON
 u.Id_porto_partenza = l.id
 LEFT JOIN task t
 ON u.id_task = t.id
 WHERE g.name != 'GSA16' and t.nome  != 'Task5.1b'
 ORDER BY Year
 LIMIT 0, 50000;"

cale = sql_fun(q_hauls)
cale = cale[which(cale$Lon_Cala != 0),]

cale = cale[!is.na(cale$Lat_Cala) & cale$Lat_Cala > 0,]
```

```
cale = cale[!is.na(cale$Lat_salpa) & cale$Lat_salpa > 0,]
# calcluate haul midpoints - geosphere::midPoint()
pp = midPoint(SpatialPoints(cale[,c("Lon_Cala", "Lat_Cala")]),
              SpatialPoints(cale[,c("Lon_Cala", "Lat_salpa")]))
cale$lon = pp[,1]
cale$lat = pp[,2]
colnames(cale)[13]<- "depth"

# Marine turtles
q_tarta = "SELECT
op.id_uscita as id_uscita,
op.id as id_cala,
g.name as gsa,
l.nome as harbour,
v.nome as Vessel,
Date(datetime_cala) as Date, YEAR(datetime_cala) as Year,
MONTH(datetime_cala) as month, TIME(datetime_cala) as Time,
op.COG_Cala as route,
op.Lat_Cala, op.Lon_Cala,
op.Lat_Salpa, op.Lon_Salpa,
op.Velocita_Traina_nm_h as speed, op.Prof_Cala_m as depth,
TIMEDIFF(TIME(op.datetime_salpa ), TIME(op.datetime_cala)) as duration,
b.scala as beaufort_scale, b.velocita_vento_nds as velocita_vento_nds,
op.mitigazione, # da vedere con Sara
sp.Nome_scientifico as specie, count(tc.ID_Specie) as Specimens
FROM tartarughe_cattura tc
LEFT JOIN specie sp ON tc.ID_Specie = sp.id
LEFT JOIN operazioni_pesca op ON tc.id_cala = op.id
LEFT JOIN uscite u ON op.id_uscita = u.id
LEFT JOIN localita l on u.Id_porto_partenza = l.id
LEFT JOIN task on u.id_task = task.id
LEFT JOIN vessel v ON u.id_vessel_principale = v.id
LEFT JOIN gsa g ON op.gsa_salpa = g.id
LEFT JOIN beaufort b ON op.id_beaufort_cala = b.id
WHERE u.id_task = 1 AND g.name != 'GSA16'
GROUP BY tc.id_cala, tc.id_specie
ORDER BY  Date(datetime_cala)
LIMIT 0, 20000;"
tarta = sql_fun(q_tarta)
tarta = dcast(tarta, id_cala ~ specie, fun.aggregate = sum, value.var = "Specimens")
colnames(tarta)[2:ncol(tarta)] <- gsub(" ", "_", colnames(tarta)[2:ncol(tarta)])

# Elasmobranchs
q_elasmo = "#Select * FROM elasmobranchi_cattura;
SELECT
op.id_uscita as id_uscita,
op.id as id_cala,
g.name as gsa,
l.nome as harbour,
v.nome as Vessel,
Date(datetime_cala) as Date, YEAR(datetime_cala) as Year,
MONTH(datetime_cala) as month, TIME(datetime_cala) as Time,
op.COG_Cala as route,
op.Lat_Cala, op.Lon_Cala,
op.Lat_Salpa, op.Lon_Salpa,
op.Velocita_Traina_nm_h as speed, op.Prof_Cala_m as depth,
TIMEDIFF(TIME(op.datetime_salpa ), TIME(op.datetime_cala)) as duration,
b.scala as beaufort_scale, b.velocita_vento_nds as velocita_vento_nds,
op.mitigazione, # da vedere con Sara
sp.Nome_scientifico as specie, count(ec.id_specie) as Specimens
FROM elasmobranchi_cattura ec
LEFT JOIN specie sp ON ec.id_specie = sp.id
LEFT JOIN operazioni_pesca op ON ec.id_cala = op.id
LEFT JOIN uscite u ON op.id_uscita = u.id
LEFT JOIN localita l on u.Id_porto_partenza = l.id
LEFT JOIN task on u.id_task = task.id
LEFT JOIN vessel v ON u.id_vessel_principale = v.id
```

```
LEFT JOIN gsa g ON op.gsa_salpa = g.id
LEFT JOIN beaufort b ON op.id_beaufort_cala = b.id
WHERE u.id_task = 1 AND g.name != 'GSA16'
GROUP BY ec.id_cala, ec.id_specie
ORDER BY  Date(datetime_cala)
LIMIT 0, 20000;"
elasmo = sql_fun(q_elasmo)
elasmo = dcast(elasmo, id_cala ~ specie, fun.aggregate = sum, value.var = "Specimens")
colnames(elasmo)[2:ncol(elasmo)] <- gsub(" ", "_", colnames(elasmo)[2:ncol(elasmo)])


# Dolphins
q_delfi = "SELECT
op.id_uscita as id_uscita,
op.id as id_cala,
g.name as gsa,
l.nome as harbour,
v.nome as Vessel,
Date(datetime_cala) as Date, YEAR(datetime_cala) as Year,
MONTH(datetime_cala) as month, TIME(datetime_cala) as Time,
op.COG_Cala as route,
op.Lat_Cala, op.Lon_Cala,
op.Lat_Salpa, op.Lon_Salpa,
op.Velocita_Traina_nm_h as speed, op.Prof_Cala_m as depth,
TIMEDIFF(TIME(op.datetime_salpa ), TIME(op.datetime_cala)) as duration,
b.scala as beaufort_scale, b.velocita_vento_nds as velocita_vento_nds,
op.mitigazione, # da vedere con Sara
sp.Nome_scientifico as specie, count(cc.id_specie) as Specimens
FROM cetacei_cattura cc
LEFT JOIN specie sp ON cc.id_specie = sp.id
LEFT JOIN operazioni_pesca op ON cc.id_cala = op.id
LEFT JOIN uscite u ON op.id_uscita = u.id
LEFT JOIN localita l on u.Id_porto_partenza = l.id
LEFT JOIN task on u.id_task = task.id
LEFT JOIN vessel v ON u.id_vessel_principale = v.id
LEFT JOIN gsa g ON op.gsa_salpa = g.id
LEFT JOIN beaufort b ON op.id_beaufort_cala = b.id
LEFT JOIN task t on u.id_task= t.id
WHERE g.name != 'GSA16' and t.nome  != 'Task5.1b' and l.nome !='Sciacca'
AND check_catch_real !=0
GROUP BY cc.id_cala, cc.id_specie
ORDER BY  Date(datetime_cala)
LIMIT 0, 20000;"

delfi = sql_fun(q_delfi)
delfi = dcast(delfi, id_cala ~ specie, fun.aggregate = sum, value.var = "Specimens")
colnames(delfi)[2:ncol(delfi)] <- gsub(" ", "_", colnames(delfi)[2:ncol(delfi)])

# Create a unique dataset:
ydat = merge(merge(merge(cale, elasmo, by = "id_cala", all.x = T),
    tarta, by = "id_cala",all.x = T), delfi, by = "id_cala",all.x = T)

colnames(ydat)[4]<- "year"
ydat$quarter = quarter(ydat$date)
ydat$month = month(ydat$date)

# Variables selection:
ydat<- ydat[, c("id_cala", "year", "quarter", "month", "datetime_cala", "lon", "lat", "depth",
                "(ND)_RAJIFORME",             "(ND)_SQUALIFORME",
                "Aetomylaeus_bovinus",        "Alopias_vulpinus",
                "Carcharhinus_plumbeus",      "Dasyatis_pastinaca",
                "Mustelus_asterias",          "Mustelus_mustelus",
                "Mustelus_punctulatus",       "Mustelus_spp",
                "Myliobatis_aquila",          "Prionace_glauca",
                "Pteroplatytrygon_violacea",  "Raja_asterias",
                "Raja_clavata",               "Raja_miraletus",
                "Scyliorhinus_canicula",      "Scyliorhinus_stellaris",
```

```
                "Squalus_acanthias",          "Torpedo_marmorata",
                "(ND)_TARTARUGA",
                "Caretta_caretta",            "Tursiops_truncatus")]

# Save:
saveRDS(ydat, "xxxxxx/results/ydat.rData")
# load grid - a SpatialPolygonDataFrame

wgs = "+proj=longlat +datum=WGS84 +no_defs +ellps=WGS84 +towgs84=0,0,0"
grid = readOGR("xxxxxxx/grid_base_5nm.shp")
proj4string(grid) = wgs
grid@data = data.frame(id_cell = grid@data$id)
xcent = gCentroid(grid, byid = T)
grid@data$lon = xcent@coords[,1]
grid@data$lat = xcent@coords[,2]
colnames(grid@data) = c("ID_CELL", "LON", "LAT")

# Maps - Datasets preparation ####
#dolphins
dolphins<- ydat %>% dplyr::select(c(ID_CELL,id_cala, year, lon, lat, Tursiops_truncatus))
dolphins[is.na(dolphins)] <- 0
dolphins<- dolphins%>% dplyr::select(ID_CELL, Tursiops_truncatus) %>%
  group_by(ID_CELL) %>% summarize(dolphins= sum(Tursiops_truncatus))
grid_dolphins = grid
grid_dolphins = sp::merge(grid_dolphins, dolphins, by = "ID_CELL", all.x = T)
grid_dolphins@data[is.na(grid_dolphins@data)] <- 0



#sharks
sharks<- ydat[,c("ID_CELL", "id_cala","year", "lon", "lat", "(ND)_SQUALIFORME",
                 "Alopias_vulpinus", "Carcharhinus_plumbeus","Mustelus_asterias",
                 "Mustelus_mustelus",
                 "Mustelus_punctulatus", "Mustelus_spp", "Prionace_glauca", "Scyliorhinus_canicula",
                 "Scyliorhinus_stellaris","Squalus_acanthias")]
sharks[is.na(sharks)] <- 0
sharks<- melt(sharks, id.vars = c("ID_CELL","id_cala", "year", "lon", "lat"))
sharks<- sharks%>% dplyr::select(ID_CELL, value) %>%
  group_by(ID_CELL) %>% summarize(sharks= sum(value))
grid_sharks = grid
grid_sharks = sp::merge(grid_sharks, sharks, by = "ID_CELL", all.x = T)
grid_sharks@data[is.na(grid_sharks@data)] <- 0

batoids<- ydat[,c("ID_CELL","id_cala", "year", "lon", "lat",
                  "(ND)_RAJIFORME", "Aetomylaeus_bovinus", "Dasyatis_pastinaca",
                  "Myliobatis_aquila", "Pteroplatytrygon_violacea", "Raja_asterias",
                  "Raja_clavata", "Raja_miraletus", "Torpedo_marmorata" )]
batoids[is.na(batoids)] <- 0
batoids<- melt(batoids, id.vars = c("ID_CELL","id_cala", "year", "lon", "lat"))
batoids<- batoids%>% dplyr::select(ID_CELL, value) %>%
  group_by(ID_CELL) %>% summarize(batoids= sum(value))
grid_batoids = grid
grid_batoids = sp::merge(grid_batoids, batoids, by = "ID_CELL", all.x = T)
grid_batoids@data[is.na(grid_batoids@data)] <- 0



turtles<- ydat[, c("ID_CELL","id_cala", "year", "lon", "lat", "(ND)_TARTARUGA",
                   "Caretta_caretta")]
turtles[is.na(turtles)] <- 0
turtles<- melt(turtles, id.vars = c("ID_CELL","id_cala", "year", "lon", "lat"))
turtles<- turtles%>% dplyr::select(ID_CELL, value) %>%
  group_by(ID_CELL) %>% summarize(turtles= sum(value))
grid_turtles = grid
grid_turtles = sp::merge(grid_turtles, turtles, by = "ID_CELL", all.x = T)
grid_turtles@data[is.na(grid_turtles@data)] <- 0
```

```r
#Spatial grids: ####
# 1) grid_dolphins
# 2) grid_sharks
# 3) grid_batoids
# 4) grid_turtles

gd<- st_as_sf(grid_dolphins)
gs<- st_as_sf(grid_sharks)
gb<- st_as_sf(grid_batoids)
gt<- st_as_sf(grid_turtles)



# Plots ####

#dolphins
dolphins_mycol<- c("#FFFFFF", sf.colors(2))
range(gd$dolphins)
dp_values<- c(0,1,2)
dp_vec<- findInterval(gd$dolphins, dp_values, left.open = T)
dp_vec<- dp_vec+1
dp_Intervals<- c("0","1","2")
dpSpecimens<- dp_Intervals[dp_vec]
dpSpecimens<- factor(dpSpecimens, levels = c("0","1","2"))
gd$Specimens<- dpSpecimens
dp<- ggplot(gd) + geom_sf(aes(fill=Specimens), colour =NA) +
  geom_sf(data = Map1, fill = "gray", colour= "grey25")+
  xlim(c(12,16)) + ylim(c(42,46))+
  scale_fill_manual(values = dolphins_mycol, name= "Specimens") +
  ggtitle("Dolphins") +
  theme(plot.title = element_text( face = "bold"))


#sharks
sharks_mycol<- c("#FFFFFF", sf.colors(9))
range(gs$sharks)
sp_values<- c(0,10,20,30, 40,50, 100, 150, 200, 250)
sharks_vec<- findInterval(gs$sharks, sp_values, left.open = T)
sharks_vec<- sharks_vec+1
sk_Intervals<- c("0", "1-10", "10-20", "20-30", "30-40",
                 "40-50", "50-100", "100-150", "150-200", ">200")
skSpecimens<- sk_Intervals[sharks_vec]
skSpecimens<- factor(skSpecimens, levels = c("0", "1-10", "10-20", "20-30", "30-40",
                                            "40-50", "50-100", "100-150", "150-200", ">200"))
gs$Specimens<- skSpecimens
#plot
sp<- ggplot(gs) + geom_sf(aes(fill=Specimens), colour=NA) +
  geom_sf(data = Map1, fill = "gray", colour= "grey25")+
  xlim(c(12,16)) + ylim(c(42,46)) +
  scale_fill_manual(values = sharks_mycol, name= "Specimens") +
  ggtitle("Sharks") +
  theme(plot.title = element_text( face = "bold"))

#batoids
batoids_mycol<- c("#FFFFFF", sf.colors(9))
range(gb$batoids)
bp_values<- c(0,10,20,30, 40,50, 100, 150, 200)
batoids_vec<- findInterval(gb$batoids, bp_values, left.open = T)
batoids_vec<- batoids_vec+1
bat_Intervals<- c("0", "1-10", "10-20", "20-30", "30-40",
                  "40-50", "50-100", "100-150", "150-200")
batSpecimens<- bat_Intervals[batoids_vec]
batSpecimens<- factor(batSpecimens, levels = c("0", "1-10", "10-20", "20-30", "30-40",
                                              "40-50", "50-100", "100-150", "150-200"))
gb$Specimens<- batSpecimens
#plot
bp<- ggplot(gb) + geom_sf(aes(fill=Specimens), colour= NA) +
```

```
  geom_sf(data = Map1, fill = "gray", colour= "grey25")+
  xlim(c(12,16)) + ylim(c(42,46)) +
  scale_fill_manual(values = batoids_mycol, name= "Specimens") +
  ggtitle("Skates and rays") +
  theme(plot.title = element_text( face = "bold"))



#turtles
mycol<- c("#FFFFFF", sf.colors(5))
range(gt$turtles)
tp_values<- c(0,5,10,20,30,40,50,60,70)
i_vec<- findInterval(gt$turtles, tp_values, left.open = T)
i_vec<- i_vec+1
Intervals<- c("0", "1-5", "5-10", "10-20", "20-30", "30-40", "40-50", "50-60", "60-70")
Specimens<- Intervals[i_vec]
Specimens<- factor(Specimens, levels = c("0", "1-5", "5-10", "10-20", "20-30", "30-40", "40-50",
"50-60", "60-70"))
gt$Interval<- Interval
#plot
tp<- ggplot(gt) + geom_sf(aes(fill=Specimens), colour=NA) +
  geom_sf(data = Map1, fill = "gray", colour= "grey25")+
  xlim(c(12,16)) + ylim(c(42,46))  +
  scale_fill_manual(values = mycol, name= "Specimens") +
  ggtitle("Sea turtles") +
  theme(plot.title = element_text( face = "bold"))


# Full plot
grid.arrange(dp, tp, sp, bp, ncol=2)
```
